# Construction of a MicroRNA-mRNA Network Underlying Decidualized Endometriotic Cyst Stromal Cells Using Bioinformatics Analysis

**DOI:** 10.1155/2020/9246868

**Published:** 2020-08-19

**Authors:** Junzui Li, Bin Zhao, Cui Yang, Qionghua Chen

**Affiliations:** School of Medicine, Xiamen University, Xiamen, Fujian, China

## Abstract

**Background:**

Decidualization of ectopic endometrium often leads to the extensive proliferation of local tissue and is easily misdiagnosed as malignant tumors. The study is aimed at constructing a microRNA- (miRNA-) mRNA network underlying decidualized endometriotic cyst stromal cells (ECSCs).

**Methods:**

All data were collected from the Gene Expression Omnibus (GEO) database. Firstly, the differentially expressed genes (DEGs, adj. *P*‐Val < 0.05, | log FC | ≥1) and miRNAs (DEMs, *P*‐Val < 0.05, ∣log FC | ≥1) were analyzed by the *limma* package. Secondly, we predicted the target genes (TGs) of these DEMs through the TargetScan, miRDB, and miRTarBase databases. The overlapping genes between DEGs and TGs were screened out. Thirdly, the Kyoto Encyclopedia of Genes and Genomes (KEGG) pathway and Gene Ontology (GO) enrichment analyses of the overlapping genes were performed for integrated discovery, visualization, and annotation. Then, the protein-protein interaction (PPI) network of the overlapping genes was conducted by the STRING database. Finally, we combined the PPI network and the miRNA-mRNA pairs to build a miRNA-mRNA network.

**Results:**

There are 29 DEMs and 523 DEGs. Fourteen overlapping genes were screened out, and these genes were significantly enriched in metabolism and immunity. What is more, a miRNA-mRNA network, including 14 mRNAs and 9 miRNAs, was successfully constructed.

**Conclusions:**

Taken together, the miRNA-mRNA regulatory networks described in this study may provide new insights in the decidualization of ECSCs, suggesting further investigations in novel pathogenic mechanisms.

## 1. Introduction

Endometriosis (EMS) is defined as the presence of endometrial glands and stroma in an abnormal or ectopic location outside the uterine cavity. It occurs in approximately 6–10% of reproductive aged women and is present in 20–50% in women with infertility and 71–87% in women with chronic pelvic pain [1]. Meanwhile, the decidualization of endometrial tissue during pregnancy plays an accurate role in the regulation of blastocyst implantation, placenta formation, and maintenance of normal pregnancy. Defects of decidualization can affect trophoblast invasion of the endometrium and may lead to infertility, recurrent spontaneous abortion (RSA), intrauterine growth retardation (IUGR), preeclampsia, premature birth, and other diseases [2]. Importantly, as to patients with EMS, ectopic endometrial decidualization during pregnancy can lead to significant growth of local tissue, which is easily misdiagnosed as malignant tumors [3]. Therefore, understanding the molecular biological mechanism of ectopic endometrium decidualization is helpful for the differential diagnosis of these two diseases.

As we all know, decidualization is a physiological process involving the function and morphological changes of endometrial stromal cells and the reconstruction of extracellular matrix [4]. However, in EMS, the decidualization of the endometrium is a heterogeneous pathological process, including the decidualization of the eutopic endometrium and the decidualization of the ectopic endometrium. Previous studies have shown that the process of endometrial decidualization in patients with EMS may involve the regulation of multiple genes and cell signaling pathways [5, 6]. Su et al. suggested decreasing Notch pathway signaling in the endometrium of women with EMS can impair decidualization of the eutopic endometrium [7]. Cho et al. pointed out that reducing Akt activity and FOXO3a expression in human endometrial stromal cells can inhibit decidual formation of the eutopic endometrium [8]. These studies on decidualization focused on the eutopic endometrial tissue. However, there are few studies on the mechanism of ectopic endometrial decidualization in EMS patients.

Nowadays, many microarray studies have shown that miRNAs are differentially expressed in eutopic endometrial tissues with EMS and ectopic endometrial tissues with EMS [9]. However, so far, there are few reports on genes, miRNAs, and cell signaling pathways related to ectopic endometrial decidualization. The mechanism of these differentially expressed miRNAs in the process of ectopic endometrial decidualization remains unclear. Therefore, the research on miRNAs in the pathogenesis of EMS will be of great significance. As to patients with EMS, potential important candidate genes, miRNAs, and signaling pathways related to ectopic endometrial decidualization need to be identified. Understanding the physiological process of ectopic endometrial decidualization is helpful for the early diagnosis of EMS and the treatment of infertility caused by EMS.

**Figure 1:**
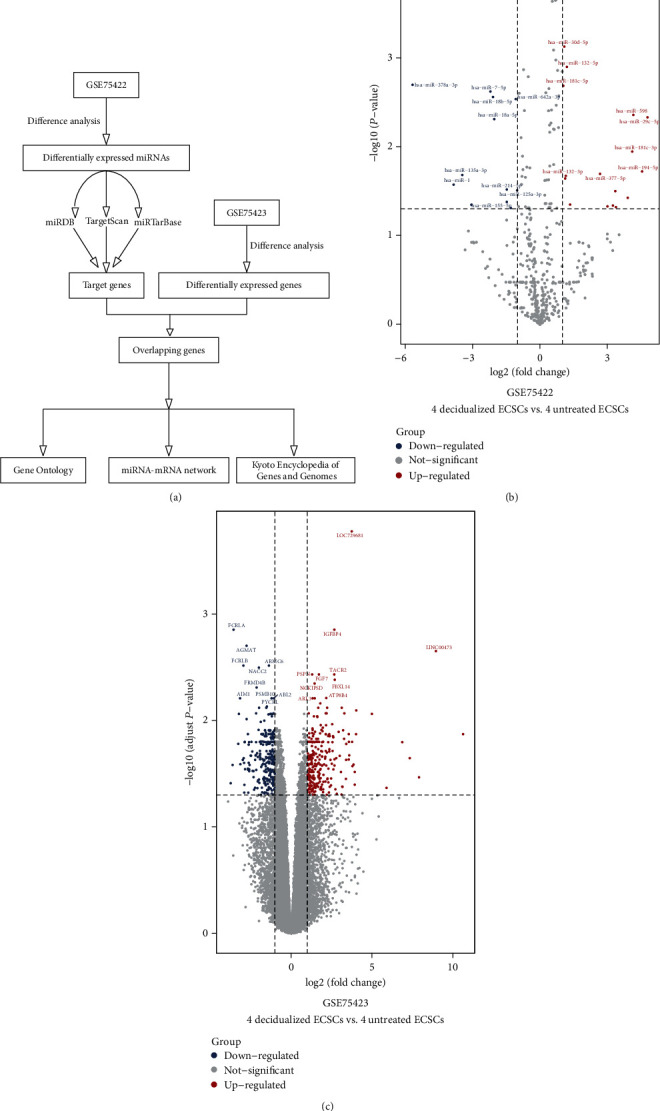
(a) The workflow of the study. (b) The volcano of the miRNAs between 4 decidualized ECSCs and 4 untreated ECSCs. (c) The volcano of the mRNAs between 4 decidualized ECSCs and 4 untreated ECSCs. Red represents upregulated miRNAs/mRNAs, and blue represents downregulated miRNAs/mRNAs. The names of the top 10 genes with the lowest *P* value among the upregulated and downregulated are visualized.

The microarray data of GSE75423 and GSE75422 have been previously used to reveal the mRNA and miRNA expression profiles in decidualized and nondecidualized endometriotic cyst stromal cells (ECSCs) [9]. Herein, we constructed a miRNA-mRNA network and performed Kyoto Encyclopedia of Genes and Genomes (KEGG) pathway and Gene Ontology (GO) enrichment analyses. This study is aimed at identifying the miRNA-mRNA network in decidualized and nondecidualized ECSCs. The workflow of this study is shown in Figure 1(a).

## 2. Materials and Methods

### 2.1. Microarray Data

We use the keywords of “endometriosis” and “decidualization” to retrieve data sets about ectopic endometriosis and decidualization in GEO database, which is created and maintained by NCBI. Then, the microarray data of GSE75423 (mRNAs, 4 untreated ECSCs and 4 decidualized ECSCs; Platforms: GPL13497, Agilent-026652 Whole Human Genome Microarray 4x44K v2) and GSE75422 (miRNAs, 4 untreated ECSCs and 4 decidualized ECSCs; Platforms: GPL18402, Agilent-046064 Unrestricted_Human_miRNA_V19.0 Microarray) were collected and downloaded for further analysis.

### 2.2. Identification of DEMs and DEGs

*limma* is a package that offers a comprehensive solution for the analysis of gene expression data and used for differentially expressed gene detection [10]. Herein, our datasets were normalized and analyzed by *limma* package built in R software. As to DEGs, the adj. *P*‐Val and |log FC| were set at <0.05 and ≥1 (decidualized ECSCs vs. untreated ECSCs). As to DEMs, the *P*‐Val and |log FC| were set at <0.05 and ≥1. Finally, the DEMs and DEGs were used in the next analysis.

### 2.3. miRNA Target Gene Prediction

The miRNA-mRNA pairs were predicted via miRDB (version 7.0; http://mirdb.org/), miRTarBase (http://mirtarbase.mbc.nctu.edu.tw/index.html) and TargetScan (Version 7.2; http://targetscan.org/vert_72/) databases. Genes that appeared in all three databases were regarded as target genes of DEMs (TGs). miRDB is an online database for miRNA target prediction and functional annotations. All the targets in miRDB were predicted by a bioinformatics tool, MirTarget, which was developed for analyzing thousands of miRNA-target interactions from high-throughput sequencing experiments. miRTarBase is a database of experimentally validated microRNA targets. TargetScan predicts biological targets of miRNAs by searching for the presence of conserved 8 mer, 7 mer, and 6 mer sites that match the seed region of each miRNA.

### 2.4. Screening of Overlapping Genes

By comparing the predicted TGs of DEMs with DEGs, only the overlapping genes and their interaction pairs were selected and be used to construct a miRNA-mRNA network.

### 2.5. Go and KEGG Pathway Enrichment Analysis of Overlapping Genes

GO is widely used in annotating genes, gene products, and sequences. KEGG is a comprehensive database for biological interpretation of genome sequences and other high-throughput data. In order to depict the features of the overlapping genes, the GO and KEGG pathway enrichment analysis of overlapping genes were performed by clusterProfiler R package with the criterion: *P* value < 0.05.

### 2.6. Construction of miRNA-mRNA Network

The online database of STRING (https://string-db.org/) was applied to assess the PPI containing direct (physical) and indirect (functional) associations. We uploaded the overlapping genes to STRING database, and the PPI networks were visualized by Cytoscape software. The minimum required interaction score in STRING Datasets is 0.4. Furthermore, we also used Cytoscape software to visualize the miRNA-mRNA pairs. Lastly, we combined the PPI network and the miRNA-mRNA pairs to build a miRNA-mRNA network.

## 3. Results

### 3.1. Identification of DEMs and DEGs

**Figure 2:**
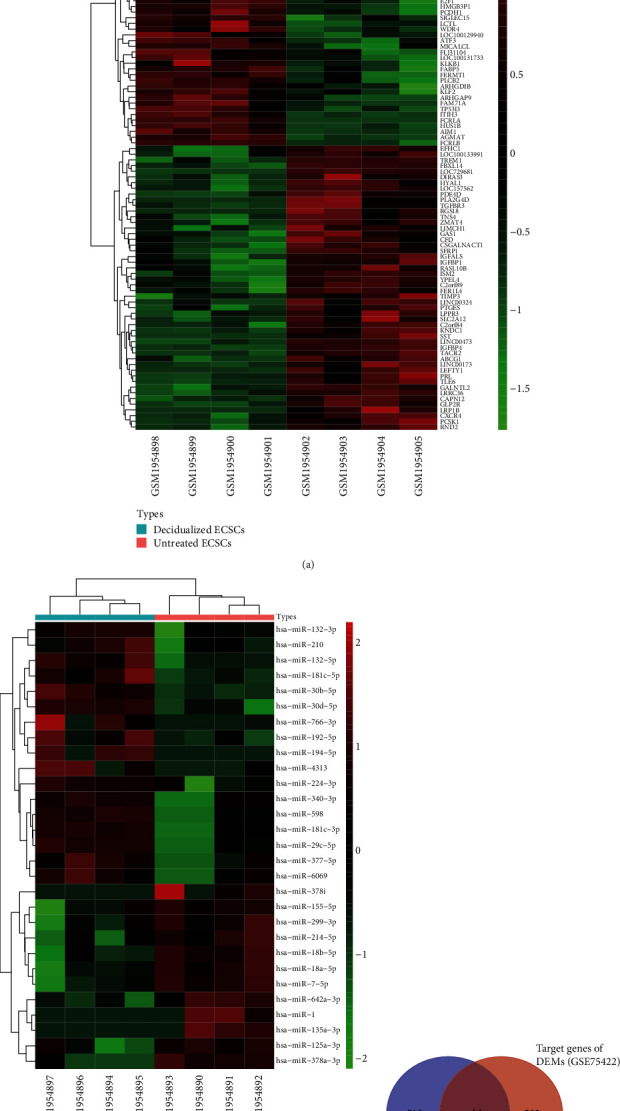
(a) Hierarchical clustering heat map of top 50 DEMs. (b) Hierarchical clustering heat map of 29 DEMs. Red indicates the upregulated expression of DEMs/DEGs. Green indicates the downregulated expression of DEMs/DEGs. (c) The overlapping genes of the DEGs and target genes of DEMs.

As shown in Figure 1(b), 1(c), 2(a), and 2(b), there were 2006 miRNAs in GSE75422 data sets, and then 29 DEMs were screened out (17 upregulated miRNAs and 12 downregulated miRNAs in decidualized ECSCs). There were 21754 mRNAs in GSE75423 data sets, and then, 523 DEGs were screened out (272 upregulated mRNAs and 251 down-regulated mRNAs in decidualized ECSCs).

### 3.2. miRNA Target Gene Prediction

Through three online databases, twenty-two miRNAs related to 606 mRNA pairs were obtained (as shown in the supplement (see available here)).

### 3.3. Screening of Overlapping Genes

As shown in Figure 2(c) and Table 1, fourteen overlapping genes were screened out and a miRNA-mRNA network was constructed. Among the miRNA-mRNA regulatory networks, some mRNAs, including *TRPS1, IGSF8, IRF2BP2, IGF1R, CELSR3* and *PDE4D*, were upregulated in decidualized ECSCs, and other mRNAs, including *MYBL2*, *PDCL3*, *KLF6*, *FUS*, *PIP4K2A*, *ASXL1*, *POLE4*, and *DDAH1*, were downregulated in decidualized ECSCs. As to miRNAs, these miRNAs, including hsa-miR-30b-5p, hsa-miR-766-3p, hsa-miR-181c-5p, hsa-miR-766-3p, hsa-miR-30b-5p, hsa-miR-7-5p, hsa-miR-7-5p, and hsa-miR-30b-5p, were upregulated in decidualized ECSCs, and some miRNAs, including hsa-miR-155-5p, hsa-miR-7-5p, hsa-miR-155-5p, hsa-miR-378a-3p, hsa-miR-30b-5p, and hsa-miR-18a-5p, were downregulated in decidualized ECSCs.

### 3.4. Go and KEGG Pathway Enrichment Analysis of Overlapping Genes

**Figure 3:**
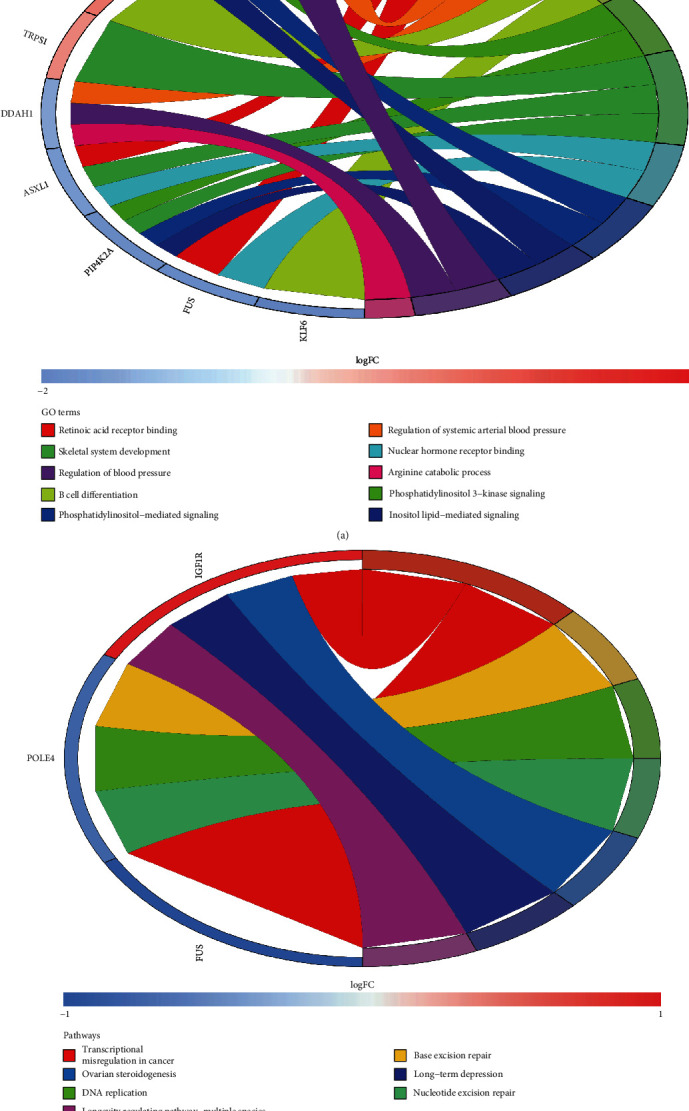
(a) GO enrichment analysis of overlapping genes. (b) KEGG pathway enrichment analysis of overlapping genes.

Through analysis, one hundred and sixty-six GO terms were enriched, and the first 10 GO terms with the most obvious enrichment are shown in Figure 3(a). GO analysis showed that 14 overlapping genes were mainly enriched in retinoic acid receptor binding, regulation of systemic arterial blood pressure, B cell differentiation, phosphatidylinositol 3-kinase signaling, skeletal system development, nuclear hormone receptor binding, phosphatidylinositol-mediated signaling, inositol lipid-mediated signaling, and regulation of blood pressure. Moreover, seven pathways were enriched. KEGG pathway enrichment analysis indicated 14 overlapping genes were mainly enriched in transcriptional misregulation in cancer, base excision repair, DNA replication, nucleotide excision repair, ovarian steroidogenesis, long-term depression, and longevity regulating pathway-multiple species, as shown in Figure 3(b) and Table 2. In summary, the Go and KEGG pathway enrichment analyses of overlapping genes were significantly enriched in metabolism (retinoic acid receptor binding, phosphatidylinositol-mediated signaling, inositol lipid-mediated signaling, ovarian steroidogenesis and arginine catabolic process, etc.) and immunity (such as B cell differentiation).

### 3.5. Construction of a miRNA-mRNA Network

**Figure 4:**
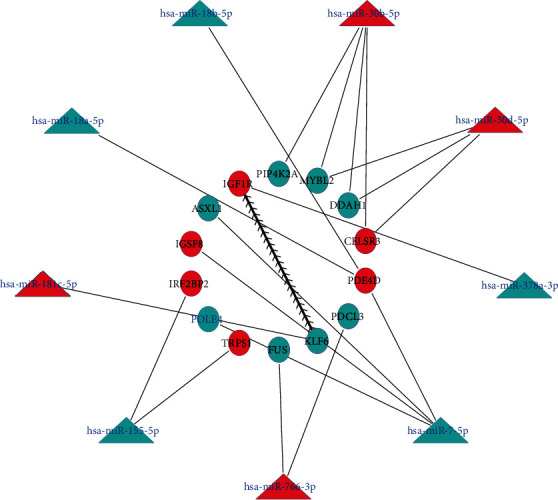
A miRNA-mRNA network. Circle represents mRNA and triangle represents miRNA. The lines represent the connection within them. It is worth noting that there are only two genes (IGF1R and KLF6) in the PPI network. Red represents upregulated miRNAs/mRNAs, and blue represents downregulated miRNAs/mRNAs.

As shown in Figure 4, a total of 2 nodes and 1 edge were mapped in the PPI network of the overlapping genes (*IGF1R* and *KLF6*). In addition, a miRNA-mRNA network, including 14 mRNAs (e.g., *IGF1R*, *DDAH1*, and *KLF6*) and 9 miRNAs (e.g., hsa-miR-378a-3p, miRNA-766-3p, and hsa-miR-7-5p), was successfully constructed.

## 4. Discussion

EMS is a common gynecological disease in women, which can lead to symptoms such as pelvic pain and female infertility. At the same time, the endometrial tissue undergoes periodic decidualization during the women's menstrual cycle. It is worth noting that the decidualization of ectopic endometrial tissue in patients with EMS is easily confused with malignant ovarian tumors, and its specific molecular biological mechanism is still unclear. Therefore, an in-depth study of the key genes and mechanisms of ectopic endometrial decidualization is of great significance for the diagnosis and treatment of EMS.

Recently, much attention has been focused on miRNAs, which can regulate gene expression at the post-transcriptional level, thereby further affecting cell proliferation, migration and invasion, signal transduction, autophagy, and apoptosis [11–13]. Meanwhile, bioinformatics, as an emerging discipline, is used to deal with genetic data and identify novel diagnosis markers [14, 15]. However, to date, no researchers have adopted this method to study the mechanism of ectopic endometrial decidualization.

In this study, we employed an integrative methodology to construct a miRNA-mRNA network and analyzed undiscovered pathways possibly regulated by those miRNAs. In our view, this innovative strategy of analysis may help to shed light on the genetic background of the disease, suggesting further molecular investigations in novel pathogenic mechanisms. Firstly, we selected 29 DEMs and 523 DEGs as our subsequent research object. Then, we constructed a miRNA-mRNA network and found 14 overlapping genes in miRNA-mRNA network may participate in the process of decidualization of ECSCs through metabolism (e.g., retinoic acid receptor binding, phosphatidylinositol-mediated signaling, inositol lipid-mediated signaling, ovarian steroidogenesis, and arginine catabolic process) and immunity (such as B cell differentiation). Many immunological factors are known to contribute significantly to the pathogenesis and pathophysiology of EMS, and both chronic local inflammation and autoantibodies in EMS share numerous similarities with autoimmune diseases (AD). Previous studies have shown that soluble chemoattractant proteins expressed in ectopic tissues of patients with EMS can recruit innate immune cells (such as neutrophils, natural killer cells, and macrophages) to accumulate in ectopic endometrial tissues [16, 17]. However, the relationship between decidualization of ectopic endometrium and immunity has not been reported. Our results further clarify the role of immunity in EMS and suggest that the immune system may play an important role in the decidualization of the ectopic endometrium.

In terms of metabolism, many studies have reported that decidualization of eutopic endometrium is related to metabolism, such as PKM2 and BPA [18, 19]. However, molecular biological processes related to metabolism in the process of decidualization of ECSCs have not been reported yet. Our results suggest that decidualization of ectopic endometrium may also be related to retinoic acid receptor (RAR). It is well known that RAR can regulate gene expression after binding with retinoic acid (RA) to maintain tissue differentiation. In endometriotic stromal cells, decreased expression of RAR leads to apoptosis and reduces cell survival [20]. At the same time, in the process of decidualization, there is apoptosis in decidual cells. Therefore, this provides us with some enlightenment. We speculated that RAR may be related to the presence of decidual cells in the process of decidualization of ECSCs.

In the miRNA-mRNA network, miRNA-30d-5p, miRNA-30b-5p, miRNA-181c-5p, and miRNA-766-3p were highly expressed in decidual ECSCs, while *PIP4K2A*, *MYBL2*, *DDAH1*, *KLF6*, *FUS*, and *PDCL3*, the target genes of those miRNAs were low expressed in decidual ECSCs. What is more, miRNA-378a-3p, miRNA-7-5p, miRNA-55-5p, miRNA-18a-5p, and miRNA-18b-5p were low expressed in decidual ECSCs, while *IGF1R*, *PDE4D*, *IGSF8*, *IRF2BP2*, and *TRPS1*, the target genes of those miRNAs were highly expressed in decidual ECSCs. This result showed that these miRNAs may target some mRNAs that correspond to them and play a role in decidualization of ECSCs.

Among the 14 overlapping genes, we found 6 downregulated mRNAs and 8 upregulated mRNAs in decidualized ECSCs. Some of them have been reported to play important roles in the development of tumor proliferation, apoptosis, and metastasis, such as *IGF1R* [21], *MYBL2* [22], *POLE* [23], and *DDAH1* [24]. Some of them were involved in various inflammatory and immune responses, such as *PDE4D* [25] and *IRF2BP2* [26]. It is well known that phosphodiesterases (PDEs) can hydrolyze the second messenger (cAMP) in the cell, which further plays an important role in regulating cell activities. *PDE4D* is a member of this large family of PDEs. The protein encoded by the *PDE4D* can degrade cAMP. *PDE4D* could regulate the function of cells in the inflammatory response through cAMP-dependent pathways, such as activation and proliferation of T lymphocytes, release of inflammatory factors, and aggregation of monocytes and neutrophils [27–29]. At the same time, it is worth noting that the decidual process is initiated through the cAMP signaling pathway. This further supports our prediction that *PDE4D* may participate in decidualization by regulating the inflammatory response. In addition, some genes were related to metabolism and signal transduction, such as *IRF2BP2* [30], *PIP4K2A* [31], and *KLF6* [32]. *PIP4K2A* and *KLF6* were related to adipogenesis. Meanwhile, in the process of camp-induced decidualization of ESCs, lipid increased. Therefore, our results suggested that these overlapping genes may play a role in the process of endometrial decidualization by regulating metabolism through some biological processes.

Nowadays, many studies have investigated the role of miRNAs in EMS [33, 34]. In our study, 9 miRNAs were screened out (miRNA-378a-3p, miRNA-7-5p, miRNA-55-5p, miRNA-18a-5p, and miRNA-18b-5p were low expressed in decidual ECSCs, and miRNA-30d-5p, miRNA-30b-5p, miRNA-181c-5p, and miRNA-766-3p were highly expressed in decidual ECSCs). According to previous research reports, downregulation of mir-378a-3p [35], miR-181b-5p [36], and miR-155 [37] is beneficial to the formation of decidualization. This is in agreement with our results. Therefore, we speculate that these 3 miRNAs may also be involved in decidualization of ECSCs. However, it needs to be verified by experiments. It is worth noting that the relationship between the other 6 miRNAs and decidualization or EMS has not been reported. Recent research shows that these 6 miRNAs participate in the occurrence and development of various diseases [38], such as tumor growth [39], metastasis and cell differentiation, through immunity, and metabolism [40]. This study may provide new insights for the study of these 6 miRNAs in decidualization.

## 5. Conclusion

In conclusion, through microarray of miRNA-mRNA expression profiles, we discovered the miRNAs, potentially regulated genes, and possible pathways associated with decidualization of ECSCs. The molecular roles that these dysregulated miRNAs play in EMS were not completely elucidated. Our study, however, had some obvious limitations. The further functional experiments are needed to back and validate the function of these miRNAs in the decidualization of ECSCs.

## Figures and Tables

**Table 1:** miRNA-mRNA network of overlapping genes.

miRNA	mRNA	log FC
hsa-miR-155-5p	IRF2BP2	1.278440075
hsa-miR-155-5p	TRPS1	1.005964272
hsa-miR-181c-5p	KLF6	-1.557718922
hsa-miR-18a-5p	PDE4D	3.276145824
hsa-miR-30b-5p	CELSR3	1.913555396
hsa-miR-30b-5p	DDAH1	-1.029581059
hsa-miR-30b-5p	MYBL2	-2.929273223
hsa-miR-30b-5p	PIP4K2A	-1.379812917
hsa-miR-378a-3p	IGF1R	1.382426315
hsa-miR-7-5p	ASXL1	-1.135463993
hsa-miR-7-5p	IGSF8	1.170019265
hsa-miR-7-5p	POLE4	-1.04694931
hsa-miR-766-3p	FUS	-1.404370443
hsa-miR-766-3p	PDCL3	-1.658095629

**Table 2:** Pathway enrichment analyses of overlapping genes.

ID	Description	*P* value	Gene ID
hsa05202	Transcriptional misregulation in cancer	0.007683802	IGF1R/FUS
hsa03410	Base excision repair	0.024668593	POLE4
hsa03030	DNA replication	0.026885814	POLE4
hsa03420	Nucleotide excision repair	0.034979724	POLE4
hsa04913	Ovarian steroidogenesis	0.036445299	IGF1R
hsa04730	Long-term depression	0.04447285	IGF1R
hsa04213	Longevity regulating pathway-multiple species	0.045926399	IGF1R

## Data Availability

The expression data associated with this article is available on GEO databases (https://www.ncbi.nlm.nih.gov/geo/).

## Supplementary Materials

Supplementary Materials The first table named “miRNA-mRNA pairs” is a supplement to the manuscript. The second table named “GO enrichment analysis” and the third table named “KEGG enrichment analysis” are used.
